# Neural correlates of automatic emotion regulation and their association with suicidal ideation in adolescents during the first 90-days of residential care

**DOI:** 10.1038/s41398-023-02723-9

**Published:** 2024-01-23

**Authors:** Matthew Dobbertin, Karina S. Blair, Joseph Aloi, Sahil Bajaj, Johannah Bashford-Largo, Avantika Mathur, Ru Zhang, Erin Carollo, Amanda Schwartz, Jaimie Elowsky, J. L. Ringle, Patrick Tyler, R. James Blair

**Affiliations:** 1 Center for Neurobehavioral Research, Boys Town National Research Hospital, Boys Town, NE USA; 2 Department of Psychiatry, Indiana University School of Medicine, Indianapolis, IN USA; 3 Department of Cancer Systems Imaging, MD Anderson Cancer Center, Houston, TX USA; 4 Multimodal Clinical Neuroimaging Laboratory, Institute for Human Neuroscience, Boys Town, NE USA; 5 Department of Psychology and Human Development, Peabody College, Vanderbilt University, Nashville, TN USA; 6 USC Stevens Neuroimaging and Informatics Institute, Keck School of Medicine of USC, University of Southern California, Los Angeles, CA USA; 7 Stritch School of Medicine, Loyola University Medical Center, Maywood, IL USA; 8 University of North Dakota, Grand Forks, ND USA; 9 University of Nebraska Department of Psychology, Lincoln, NE USA; 10 Child and Family Translational Research, Boys Town National Research Hospital, Boys Town, NE USA; 11 Child and Adolescent Mental Health Centre, Mental Health Services, Capital Region of Denmark, Copenhagen, Denmark

**Keywords:** Predictive markers, Human behaviour

## Abstract

Background: Suicide is the second leading cause of death for adolescents in the United States. However, relatively little is known about the forms of atypical neuro-cognitive function that are correlates of suicidal ideation (SI). One form of cognitive/affective function that, when dysfunctional, is associated with SI is emotion regulation. However, very little work has investigated the neural correlates of emotion dysregulation in adolescents with SI. Methods: Participants (*N* = 111 aged 12-18, 32 females, 31 [27.9%] reporting SI) were recruited shortly after their arrival at a residential care facility where they had been referred for behavioral and mental health problems. Daily reports of SI were collected during the participants’ first 90-days in residential care. Participants were presented with a task-fMRI measure of emotion regulation – the Affective Number Stroop task shortly after recruitment. Participants were divided into two groups matched for age, sex and IQ based on whether they demonstrated SI. Results: Participants who demonstrated SI showed increased recruitment of regions including dorsomedial prefrontal cortex/supplemental motor area and parietal cortex during task (congruent and incongruent) relative to view trials in the context of emotional relative to neutral distracters. Conclusions: Participants with SI showed increased recruitment of regions implicated in executive control during the performance of a task indexing automatic emotion regulation. Such data **might suggest** a relative inefficiency in the recruitment of these regions in individuals with SI.

## Introduction

Suicide is the second leading cause of death amongst adolescents in the US [1]. Rates of suicidal ideation (SI; defined as any verbalization, behavior, or gesture indicating suicidal thoughts or plans) are high during adolescence [2] with an estimated lifetime prevalence of SI of 12.1% in adolescents (13–18 years) [3]. There has been a recent surge in functional (at least resting state) neuroimaging studies relating to SI [4–6]; for recent review see Dobbertin et al. [7]. However, relatively little work has specifically investigated *adolescents* engaging in SI. One neuro-cognitive risk factor associated with SI and potentially suicidal behavior (SB) is emotion dysregulation [8–11]. Older adolescents/young adults who report more emotion regulation difficulties show greater SI even after accounting for depression symptoms [12]. Moreover, emotion dysregulation has been considered the core deficit of Borderline Personality Disorder, a condition associated with a highly elevated risk for non-suicidal self-injury (NSSI) and SI (e.g., [13]). However, little neuro-imaging work has considered emotion regulation in adolescents engaging in SI.

Despite the attention to emotion dysregulation, the neuro-cognitive details of emotion dysregulation in individuals with elevated SI have not been worked through. Emotion dysregulation might reflect very considerably elevated emotional responsiveness (such that intact regulatory systems are “overwhelmed”) and/or deficient emotional regulatory systems (such that emotional responses are insufficiently suppressed; for details of the model, see Supplemental Fig. 2 & [14–16]). Currently available neuroimaging data does not support the suggestion of emotional over-responsiveness in individuals with elevated SI (for a review, see [17]). Some studies even report hypo-responsiveness [18]. However, there are data indicating atypical resting state functional connectivity between the amygdala and a variety of cortical systems (e.g., [19, 20]). Such findings might reflect dysfunctional emotional regulation. Very little neuroimaging work though has directly examined emotional regulation in individuals with elevated SI. One study reported that youth with SI showed greater dorsolateral prefrontal cortex (dlPFC) responses than comparison youth during cognitive reappraisal vs. passive viewing of negative images [10]. While these data might suggest inefficient recruitment of brain regions involved in emotional regulation in youth with SI, more work is clearly needed.

Active downregulation via cognitive reappraisal is thought to involve the recruitment of regions implicated in top-down attention such that the representational focus of the emotional image is altered (dorsomedial, lateral frontal [dmFC and dlFC] and parietal cortices; [21, 22]). However, this can also occur “automatically” as a result of recruitment of regions implicated in top-down attention to task-related stimuli (these are attentionally primed) such that emotional responding to *emotional distracters* is reduced [16, 23, 24]. The current study investigated this form of “automatic” emotional regulation in adolescents with SI and adolescents without SI via the Affective Number Stroop task [16]. During performance of this task, participants either view emotional or neutral images and perform goal-directed activity (counting the number of numerals) in the context of emotional or neutral distracters (see Supplemental Figure S1**)**. Performing goal-directed activity reduces BOLD responses in emotion-relevant regions (e.g., the amygdala) to emotional distracters [16, 23].

Notably, much work investigating SI uses measures involving retrospective self-reports of SI covering varying periods of time before the assessment [6, 25]. Such measures are clearly useful. However, given that SI level shows considerable fluctuation over time [26, 27], self-reports of SI may identify trait SI rather than SI occurring in a specific period. At any single timepoint, patients may be less than forthcoming regarding suicidal thoughts and behaviors for a myriad of reasons, including fear of stigma, avoidance of hospitalization or medical treatment, or lack of insight into the severity of these thoughts and behaviors. The current study takes advantage of having a population which is heavily monitored for SI and uses the number of the *observed/recorded* SI episodes as the index of SI. Specifically, the current study examines the association of atypical neuro-cognitive emotion regulation activity, recorded shortly after the participants arrival in residential care, with SI occurring in the participants’ first 90-day window of residential care.

In line with findings using the cognitive reappraisal task [10], we predicted that adolescents with SI would show greater recruitment of regions implicated in attentional control (dlPFC but also dmFC and parietal cortex) relative to adolescents without SI when responding to task stimuli in the context of negative relative to neutral distracters. Specifically, we predicted that we would identify regions displaying Condition-by-valence-by-SI status interactions such that adolescents with SI would show greater differential responsiveness on task trials (congruent and incongruent) relative to view trials in the presence of emotional relative to neutral distracters; i.e., participants with SI should show greater BOLD responses to [emotional task trials-emotional view]-[neutral task trials-neutral view] relative to participants without SI. Note we did not make differential predictions with respect to congruent vs. incongruent trials on the basis of previous use of this task with adolescent populations (Bashford-Largo et al., [28]).

## Materials and methods

### Participants

Study included 111 youths (aged 12–18 years [*M* = 16.2 years, *SD* = 1.43]; IQ 76-134 range [M = 100.0, SD = 12.38]; 32 females). Participants were recruited after their arrival at a residential care facility (Boys Town). All had been referred to this facility for behavioral and/or mental health problems. An additional 9 participants (to the 111 study participants) were scanned on the task but excluded from analysis (due to excessive movement [>10% censored volumes at >0.5 mm motion across adjacent volumes; *N* = 4] and low response rate [*N* = 5]). Clinical characterization was completed through psychiatric interviews by licensed psychiatrists with the participant and a parent/legal guardian following standard clinical practice.

The exclusion criteria for participants in the study included pervasive developmental disorder, Tourette’s syndrome, lifetime history of psychosis, neurological disorder, head trauma, non-psychiatric medical illnesses requiring medications that may have psychotropic effects (e.g., beta-blockers, steroids), and IQ < 75. Institutional Review Board approval was acquired before data collection began. Informed consent was obtained from a parent/legal guardian and informed assent was obtained from the youth.

### Measures

#### Suicidal ideation (SI)

SI data were collected from the facility’s electronic youth records based on daily staff observations that were documented and reported to a program supervisor within 24 h. SIs were defined as any verbalization, behavior, or gesture indicating suicidal thoughts or plans. They were *not weighted by severity*. Participants received a score corresponding to their number of SIs during the 90-day period following arrival at the residential care facility.

#### Psychiatric symptom severity assessments

Psychopathology was assessed via the self-report forms of: (i) the Screen for Child Anxiety and Related Emotional Disorders (SCARED; [29]), an assessment of anxiety symptoms; (ii) the Suicide Risk Scale (SRS), a measure of suicide risks [30] and (iii) the Mood and Feelings Questionnaire (MFQ; [31]), an assessment of depression symptoms. IQ was assessed by the Wechsler Abbreviated Scale of Intelligence (WASI) [32].

### fMRI task

#### The affective number stroop fMRI task

The task was adapted from our prior work (see Supplemental Information for full details and Supplemental Fig. 1) [16, 33]. Each trial began with a fixation point presented in the middle of the screen. For the number trials, the fixation point was replaced by the first picture stimuli presented for 400 ms, followed by the numerical display presented for 400 ms, followed by the second picture display presented for 400 ms, followed by a blank stimulus for 1300 (see Supplemental Fig. 1). On incongruent trials, the Arabic numeral distracter information was inconsistent with the numerosity information (e.g., four 3 s; Supplemental Fig. 1a). On congruent trials, the Arabic numeral distracter information was consistent with the numerosity information; (e.g., three 3 s; Supplemental Fig. 1b). For view trials, there was no numerical display; the numerical display was replaced by a fixation point (see Supplemental Fig. 1c). The participant’s task, on congruent/incongruent trials, was to respond via button press according to the number of numerals (3, 4, 5, or 6) in the numerical display. Images (16 negative, 16 neutral, 16 positive) were selected from the International Affective Picture System [34].

There were two runs, each consisting of 16 presentations of each condition-by-valence combination throughout the run. In addition, 40 2500 ms fixation points were randomly presented throughout each run. Thus, overall, each participant was presented with 32 trials of each Condition-by-Valence condition. The study involved two runs each 9.63 min in length.

Data was collected between September 2016 and March 2020.

### MRI parameters

MRI data were collected using a 3 T Siemens Skyra scanner. Functional images were taken with a T2* weighted gradient echo planar imaging (EPI) sequence (repetition time [TR] = 2500 ms; echo time = 27 ms; 240 mm field of view; 94 × 94 matrix; 90^o^ flip angle). Whole-brain coverage was obtained with 43 axial slices (thickness 2.5 mm, voxel size 2.6 × 2.6 × 2.5 mm^3^). A high-resolution T1 anatomical scan (MP-RAGE, repetition time = 2200 ms; echo time = 2.48 ms; 230 mm field of view; 8^o^ flip angle; 256 × 208 matrix; thickness 1 mm; voxel size 0.9 × 0.9 × 1 mm^3^) in register with the EPI data set was obtained covering the whole brain with 176 axial slices.

### fMRI analysis: data preprocessing and individual level analysis

Functional MRI data were preprocessed and analyzed using Analysis of Functional NeuroImages (AFNI [18.2.15]) software (Cox, 1996). Both individual and group level analyses were conducted. At the individual level, functional images from the first four repetitions, collected prior to equilibrium magnetization, were discarded. The data from the two runs was concatenated. The participants’ anatomical scans were then individually registered to the Talairach and Tournoux atlas [35]. The individuals’ functional EPI data were then registered to their Talairach anatomical scan. The EPI datasets for each participant were spatially smoothed (isotropic 6 mm^3^ Gaussian kernel) to reduce variability among individuals and generate group maps. Next, the time series data were normalized by dividing the signal intensity of a voxel at each time point by the mean signal intensity of that voxel for each run and multiplying the result by 100, producing regression coefficients representing percent-signal change. Every TR on which motion exceeded 1 mm was censored.

Ten regressors were generated: view negative, congruent negative, incongruent negative, view neutral, congruent neutral, incongruent neutral, view positive, congruent positive, incongruent positive, missed/incorrect). Conditions were modeled with a gamma variate hemodynamic response function to account for the slow hemodynamic response. GLM fitting was performed with the ten regressors listed, six motion regressors, and a regressor modeling baseline drift (-polort 4). This produced a β**-**coefficient and an associated *t*-statistic for each voxel and regressor. There was no significant regressor collinearity.

### Statistical analyses

#### Clinical characteristics

Descriptive statistics were calculated for all demographic and clinical variables. For the clinical data, correlation analyses were conducted to determine the associations between the levels of SI and age, IQ and scores on the SCARED and MFQ. For sex, diagnostic status (Major Depressive Disorder [MDD], Generalized Anxiety Disorder [GAD], Conduct Disorder [CD] and Attention Deficit Hyperactivity Disorder [ADHD]) and medication prescriptions (stimulants, SSRIs and antipsychotics), the significance of group differences (males vs females, and cases vs not cases) in SI scores were examined by ANOVA. Group-based independent t-tests and chi-square analyses were also conducted to determine any differences adolescences reporting SI and those not reporting SI. Group membership was unknown to the experimenter during the experiment and during data processing.

#### Behavioral and movement data

Two 2 (Group: SI vs No SI)-by-3 (Condition: View, Congruent, Incongruent)-by-3 (Valence: Neutral, Positive, Negative) ANOVAs were performed on the error and reaction time (RT) data. With respect to movement, correlational analyses were conducted examining the extent of association between SI scores and three participant motion variables (censored volumes, average motion per volume, and maximum displacement during scanning).

#### BOLD response data

A 2 (Group: SI vs No SI)-by-3 (Condition: View, Congruent, Incongruent)-by-3 (Valence: Neutral, Positive, Negative) ANOVA was also conducted on the BOLD response data via AFNI’s 3dMVM. Correction for multiple comparisons was performed using a spatial clustering operation in AFNI’s *3dClustSim* utilizing the autocorrelation function (-acf) with 10,000 Monte Carlo simulations for a whole brain grey matter mask. The initial threshold was set at *p* = 0.001 [36, 37]. This procedure yielded a threshold of *k* = 19 voxels, which then results in a cluster-level false-positive probability of *p* < 0.05, corrected for multiple comparisons. To facilitate future meta-analytic work, effect sizes (partial eta square [*pη*²]) are reported in the Tables. Interactions were interpreted via contrast analyses using SPSS 25.0 (*p* < 0.05). The core interaction with respect to our hypothesis was the Group-by-Condition-by-Valence interaction. Two additional interactions of interest were: (i) the Group-by-Condition interaction (which would identify regions showing atypical recruitment as a function of SI in response to task demands irrespective of distracter type); and (ii) the Group-by-Valence interaction (which would identify regions showing atypical recruitment as a function of SI response to emotional relative to neutral images).

### Follow-up analyses

#### Potential confounds: psychiatric comorbidity and/or prescribed medications

Depending on the results of the clinical characteristics (i.e., significant group differences in diagnostic rates for specific psychiatric conditions or prescribed medications), the main ANOVA was repeated within AFNI (3dMVM) following the addition of a group variable corresponding to the group difference variable (e.g., cases with MDD vs. cases without MDD or cases prescribed SSRIs vs. cases not prescribed SSRIs).

## Results

### Clinical characteristics

Descriptive statistics were calculated for all demographic and clinical variables. The correlation analyses revealed significant associations between SI scores and scores on the SRS, SCARED and MFQ but not age or IQ (see Table 1). However, SI scores did not significantly differ between males and females, those with and without diagnoses of MDD, GAD, CD and ADHD or those with and without prescribed stimulant, SSRI or antipsychotic medications (though there was an association between intake SRS scores and prescription of SSRIs, see Supplemental Table S6).

**Table 1:** Relationships between demographic and clinical variables and Rankit-transformed, then normalized SI scores.

	Mean (sd)	*r* with SI	Gp: No-SI (*N* = 80)	Gp: SI (*N* = 31)	t, p
SI	0.77 (1.87)	–	− [0]	2.76 (SD = 2.69) [1–14]	–
Total SRS	58.08 (9.95)	0.342**	56.18 (9.46) [33–73]	62.78 (9.77) [42–77]	−2.80, *p* = 0.006
Hopelessness	57.19 (9.92)	0.333**	55.35 (9.72) [35–72]	61.74 (9.06) [45–78]	−2.71, *p* = 0.008
SI	55.00 (9.45)	0.383**	52.56 (8.32) [44–75]	61.04 (9.56) [44–76]	−3.95, *p* < 0.001
Negative self-evaluation	54.65 (10.11)	0.243*	53.55 (9.45) [34–73]	57.65 (11.25) [34–76]	−1.71, *p* = 0.092
Hostility	58.19 (8.12)	0.271*	57.75 (7.92) [40–77]	59.26 (8.68) [40–76]	−0.749, *p* = 0.456
Age	16.16 (1.43)	−0.042	16.23 (SD = 1.43)	15.97 (SD = 1.42)	0.86, *p* = 0.39
IQ	100.04 (12.38)	−0.068	100.45 (SD = 11.96)	98.97 (SD = 13.55)	0.56, *p* = 0.57
SCARED	18.66 (15.63)	0.260*	16.78 (SD = 13.78)	23.66 (SD = 19.11)	−2.05, *p* = 0.04
MFQ	14.03 (13.30)	0.229*	11.89 (SD = 11.60)	19.73 (SD = 15.88)	−2.84, *p* = 0.005
	*N*	t, p			*χ*^2^, *F*. *p*
Sex	32 females	1.55, *p* = 0.12	25.0% (*N* = 20)	28.7% (*N* = 12)	2.05, *p* = 0.15
MDD	18 cases	−0.57, *p* = 0.57	15.0% (*N* = 12)	19.4% (*N* = 6)	0.31, *p* = 0.58
GAD	34 cases	−1.73, *p* = 0.09	27.5% (*N* = 22)	38.7% (*N* = 12)	1.32, *p* = 0.25
CD	68 cases	1.24, *p* = 0.22	62.0% (*N* = 52)	51.6% (*N* = 16)	1.69, *p* = 0.19
ADHD	77 cases	−1.02, *p* = 0.31	67.5% (*N* = 54)	74.2% (*N* = 23)	0.47, *p* = 0.49
Stimulants	24 prescribed	−0.56, *p* = 0.58	20.0% (*N* = 16)	25.8% (*N* = 8)	0.44, *p* = 0.51
SSRIs	23 prescribed	−1.80, *p* = 0.07	16.3% (*N* = 13)	32.3% (*N* = 10)	3.49, *p* = 0.06
Antipsychotics	8 prescribed	−0.38, *p* = 0.71	7.5% (*N* = 6)	6.5% (*N* = 2)	0.04, *p* = 0.85

Key to Table 1: *SD* standard deviation (also in brackets), *significant at *p* < 0.05, *SI* suicidal ideation, *SCARED* screen for child anxiety and related emotional disorders, *MFQ* mood and feelings questionnaire, *t* independent *t* test value, *p* = *p* value (ts and ps correspond to significance of group differences with respect to sex, diagnostic status, prescribed medication status [left column] and level of SI group differences [right column]), *MDD* major depressive disorder, GAD generalized anxiety disorder, *CD* conduct disorder, *ADHD* attention deficit hyperactivity disorder.

SI was reported for 31 of the 111 participants. Groups differing according to whether the participant had or had not showed SI did not differ on any variable except SCARED and MFQ scores (see Table 1).

### Behavioral and movement data

The ANOVAs performed on the error and RT data revealed a significant main effect of task (*F* = 193.96 & 50.31; *p* < 0.001 for both); participants made more errors and were slower on incongruent relative to congruent trials. In addition, there was a main effect of the RT data for emotion (*F* = 5.395; p = 0.005); participants were significantly slower on negative relative to neutral trials (7.001; *p* < 0.01) and positive relative to neutral trials (*F* = 8.710; *p* < 0.005) (M(Neg) = 809.16 ms; M(Neu) = 797.29 ms; M(Pos) = 809.27 ms); see also Supplemental Table 1.

Volumes were censored if there was > 1.0 mm motion across adjacent volumes. Participants were excluded due to excessive motion (> 10% censored volumes; mean=0.6%, SD = 1.3%) or low response rate (< 60% responses) on the task (*N* = 11). There were no significant correlations between SI scores and censored volumes, average motion per volume, and maximum displacement during scanning within the final sample (r range = 0.086 to 0.171; ns).

### fMRI Data

Our initial analysis revealed regions showing our core Group-by-Condition-by-Valence interaction. In addition, regions were identified showing a significant Group-by-Condition interaction. No regions showed significant Group-by-Valence interactions. Regions showing significant main effects of Task and Emotion are reported in the Supplemental Material (Supplemental Table S2).

### Group-by-condition-by-valence

**Fig. 1:**
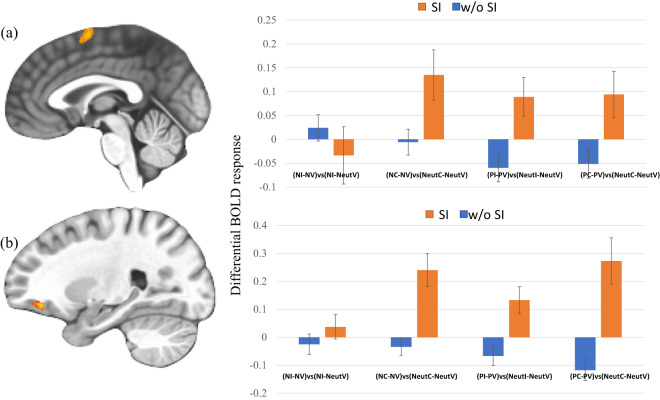
Group-by-condition-by-valence interactions. Adolescents with SI showed significant increases in activity during task relative to view trials in the context of emotional relative to neutral distracters (except for negative incongruent) within: **a** right dmPFC (x, y, z = 2, -1, 59); and **b** right IFG/OFC (x, y, z = 23, 38,-7). Key to Fig. 1: w/o & w=without and with, SI=Suicidal ideation, (NI-NV)vs(NI-NeutV)=(Negative Incongruent – Negative View)-(Neutral Incongruent – Neutral View), (NC-NV)vs(NeutC-NeutV)=(Negative Congruent – Negative View)-(Neutral Congruent – Neutral View), (PI-PV)vs(NeutI-NeutV)=(Positive Incongruent – Positive View)-(Neutral Incongruent – Neutral View), (PC-PV)vs(NeutC-NeutV)=(Positive Congruent – Positive View)-(Neutral Congruent – Neutral View).

This was observed within dmFC/supplemental motor area (SMA) and inferior frontal gyrus/orbitofrontal cortex (IFG/ OFC); see Table 2, Fig. 1. Notably, both of these regions were included within those showing a main effect of task (F(2,218) = 139.34 & 5.95, *p* < 0.001, *ηp*^2^ = 0.56 & 0.05 for dmFC/SMA and IFG/OFC respectively); see also Supplemental Table S2. Within both regions, participants with SI showed the predicted significant increase in activity during task (congruent and incongruent) relative to view trials in the context of emotional relative to neutral distracters with one exception (negative incongruent); see Fig. 1.

**Table 2:** Significant areas of activation from the 2 (Group)-by-3 (Condition)-by-3 (Valence) repeated measures ANOVA.

REGION	BA	Voxels	*X*	*Y*	*Z*	*F*-value	*ηp*²
*Group-by-Condition-by-Valence*							
R dmPFC	6	26	2	−1	59	6.37	0.06
R inferior frontal gyrus/OFC	11	18	23	38	−7	8.74	0.07
*Group-by-Condition*							
R cuneus	17/18	93	14	−88	11	14.62	0.12
R fusiform gyrus	19	109	29	−61	−10	18.25	0.14
L lingual gyrus	18	75	−37	−79	−7	15.15	0.12
L middle occipital gyrus	18	43	−7	−82	−4	14.29	0.12

Activations are effects observed in whole brain analyses significant at *p* < 0.001, corrected for multiple comparisons (significant at *p* < 0.05).

Coordinates from the Tournoux and Talairach standard brain template (TT_N27).

### Group-by-condition

**Fig. 2:**
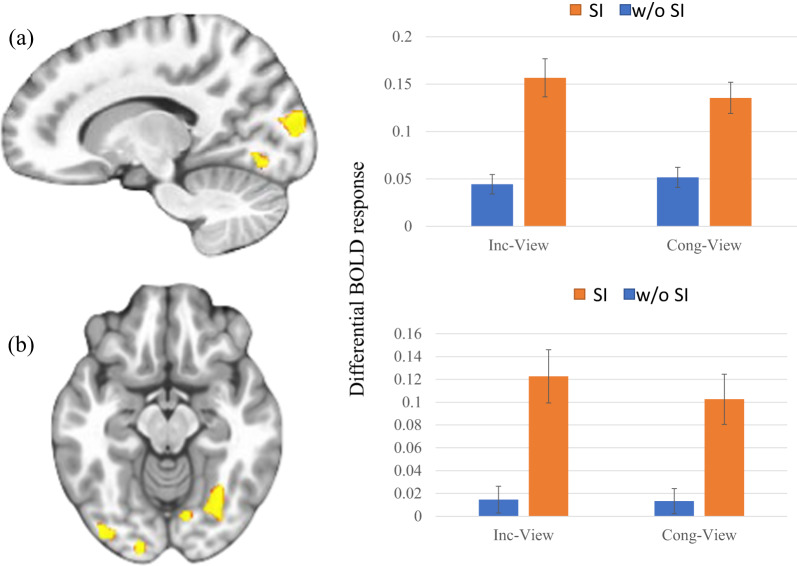
Group-by-condition interactions. Adolescents with SI showed significant increases in activity during task relative to view trials within: **a** right fusiform gyrus (x, y, z = 29, -71, -10); and **b** right cuneus (x, y, z = 14, -88, 11). Key to Fig. 1: w/o & w=without and with, SI=Suicidal ideation, Inc-View=Incongruent – View, Cong- Congruent – View.

This was observed within regions including fusiform and occipital cortex; see Table 2, Fig. 2. In all regions identified, the participants with SI showed a significantly greater increase in activity (task[incongruent and congruent trials] relative to view trials) than participants without SI.

### Potential confounds

While the majority of participants in this study presented with at least one psychiatric diagnosis, there were no significant differences in diagnostic rates of any of these diagnoses between the participants showing SI and those not showing SI (see Table 1). Similarly, prescriptions of stimulants and antipsychotic medications did not differ between the groups of participants showing, and not showing SI (see Table 1). However, there was evidence of a strong trend in group differences for prescription of SSRIs. In addition, there were significant group differences in both MFQ and SCARED scores. For this reason, the main analysis was repeated: (i) with the addition of a second group variable (SSRI prescription vs. no SSRI prescription); (ii) with the addition of MFQ as a covariate; and (iii) with the addition of SCARED as a covariate. The results of these follow-up analyses largely mirrored those of the main ANOVA.

## Discussion

The goal of this study was to determine extent of disruption of a form of “automatic” emotional regulation in participants with SI relative to participants without SI. We predicted, on the basis of previous findings examining cognitive reappraisal in adolescents with SI [10], that SI would be associated with an increased recruitment of regions implicated in attentional control (dlPFC but also dmFC and parietal cortices) during task relative to view trials in the context of emotional distracters relative to neutral distracters. This prediction was broadly supported for dmFC and inferior frontal gyrus/OFC [rather than dlPFC] but not parietal cortex. In addition, though, there was evidence that temporal and occipital regions were showing increased recruitment by adolescents with SI during task relative to view trials irrespective of the valence of the distracter context.

As noted, to our knowledge, the only previous study to directly examine the neural correlates of emotional regulation in adolescents with SI reported that youth with SI showed greater dlPFC responses than comparison youth during cognitive reappraisal vs. passive viewing negative images [10]. This could suggest inefficiency in systems engaged in emotional regulation in adolescents with SI such that they need to be recruited more strongly to achieve comparable levels of performance (there were no behavioral differences in emotion regulation in the Miller et al. [10] study). This is directly relevant to treatment interventions such as Dialectical Behavior Therapy, which focuses on improving emotion dysregulation and is one of the few treatments known to directly reduce suicide risk (MCauley et al 2018 JAMA Psychiatry). Cognitive reappraisal can be considered to rely on top-down attentional control—the non-emotional features of the visual stimulus are primed such that the response to the emotional features is weakened (e.g., [21]). “Automatic” emotional regulation occurs in paradigms such as the affective Stroop task as top-down attentional control primes task demand relevant stimulus features such that the representation of, and response to, emotional distracters is weakened [16, 23]. Our findings were directly in line with predictions. Regions involved in task performance/top-down attentional control (dmPFC and IFG/OFC) showed increased recruitment in participants with SI relative to participants without SI for task relative to view trials in the context of emotional distracters relative to neutral distracters ([emotional task trials-emotional view]-[neutral task trials-neutral view]). As such, the current data, in line with those of Miller et al. [10], are consistent with the suggestion of a relative inefficiency in systems engaged in emotional regulation in adolescents with SI; recruitment of these regions was greater in the context of emotional distracters but there was no significant effect of SI and task performance.

Three caveats to the emotion regulation argument should be briefly considered. The first of these concerns whether the current results indicate a relative inefficiency in the recruitment of dmFC/SMA and IFC/OFC (the suggestion offered above) or, instead, markers of resilience (given that the participants showing SI did not attempt suicide during the time period of the study). It is possible that the greater activity within these regions seen in the significant Group-by-Condition-by-Valence interaction reflect behavioral control processes enabling the participants to avoid suicide attempts. Future work will be necessary to determine whether participants who have recently made such attempts unsuccessfully show particularly compromised recruitment of these regions. Second, the dorsomedial region showing the significant Group-by-Condition-by-Valence interaction was slightly more posterior and the inferior frontal region slightly more inferior than would be expected from the top-down attention literature [24, 38]. Similarly, this result was not seen within parietal cortex (unless SSRI medication status was introduced into the statistical model; see Supplemental Table S3). Of course, both the dmFC/SMA and IFC/OFC regions were involved in task performance (within both regions there were significant main effects of task; incongruent & congruent > view). However, whether they were specifically involved in organizing an attentional response rather than response control cannot be determined from these data (though given the regions identified, particularly SMA, they might be more consistent with an involvement in response control, e.g., Aron et al, 2007). As such, it may be more cautious to interpret the current results as being indicative of relative inefficiency of executive functioning in context of emotional distracters in SI rather than specifically top-down attention. Third, the Group-by-Condition-by-Valence interaction was driven by particularly increased responsiveness during task performance in the context of positive distractors and during congruent trials in the context of negative distracters. There was not relatively increased responding in the context of negative incongruent trials. This was unpredicted and will be the focus of future work but might reflect breakdown in the inefficient system as a function of high salience (negative) distractors during particularly effortful (incongruent) trial performance.

Notably, no regions were identified showing a significant Group-by-Valence interaction. The clinical literature often indicates heightened threat responsiveness is a component of risk for suicidal behavior [39] and elevated startle response to uncertain threat may be associated with SI in individuals with internalizing conditions [40]. In the current study, SI was significantly associated with level of anxiety. Moreover, past exposure to abuse, which is strongly associated with increased threat responsiveness [41], is significantly associated with SI [39]. However, it should be noted that the fMRI literature does not typically support the suggestion of emotional over-responsiveness in individuals with elevated SI and/or suicidal behavior (for a review, see [17]). There have even been reports of diminished responsiveness to threat [18, 42]. This is consistent with the clinical observation of anhedonia in patients suffering from either MDD or PTSD (DSM-5). Indeed, in the emotion regulation study examining cognitive reappraisal in individuals engaging in SI, there were no indications of heightened responsiveness to threat [10]. As noted, the current study also did not indicate heightened threat or emotional responsiveness generally as a function of SI. This may reflect somewhat nebulous nature of the concept of anxiety. Anxiety in the context of SAD and PTSD is clearly associated with a particular stressor (social stimuli or trauma-related stimuli) and patients with SAD and PTSD show clear evidence of heightened threat responsiveness to these stressors [43–45]. However, anxiety in the context of GAD is more reflective of a ruminative worry [46] and the fMRI literature frequently fails to observe heightened threat responsiveness in patients with GAD (e.g., [47, 48]). Co-occurring GAD and MDD do however confer a higher risk of suicide completion [49]. Given the ruminative natures of SI, the anxiety identified by individuals with SI might be more reflective of the functional impairment associated with GAD rather than threat responsiveness specifically.

There are several caveats that should be noted with respect to the current results. First, consistent with considerable previous work [39], SI was associated with significant psychopathology. Importantly, though, the current study involved a comparison group with comparable levels of psychopathology (at least with respect to levels of specific diagnoses). Moreover, while there were group differences in both SCARED and MFQ scores, additions of these variables into the statistical model maintained the reported results. Second, given the significant psychopathology within this sample, there were relatively high levels of prescribed medications. However, there were no group differences in prescription rates for stimulant or antipsychotic medications (and only trend level group differences with respect to prescriptions of SSRIs). Moreover, the results of the main analysis held following the inclusion of SSRI medication status into the statistical model. Indeed, there was a notable increase in the number of regions identified showing a significant Group-by-Condition-by-Valence interaction within this model likely reflecting treatment effects. Third, we did not implement structured or semi-structured diagnostic interview. However, even if there was concern about the reliability of the psychiatric diagnoses, it is important to note that the goal of this work was to investigate neural signatures related to SI *across* various psychiatric diagnoses [50]. Moreover, the intake assessment was standardized—all participants were assessed on a variety of self-report measures of psychopathology including SI (Suicide Risk Scale). Fourth, scanning and SI assessment occurred on two separate days typically separated by at least a week. As such, it is possible that results may be less significant than if scanning and SI assessment had been yoked to the same day.

In conclusion, consistent with the one previous fMRI study of emotion regulation in adolescents with SI [10], participants who demonstrated SI showed an increased recruitment of regions implicated in emotion regulation as a function of task demands in the context of emotional relative to neutral distracters. These data are consistent with the suggestion of a relative inefficiency in the recruitment of these regions in participants with SI. Notably, though, given the intact behavioral performance on the current task (and lack of behavioral emotional regulation deficits in the earlier study [10], together with data from this study and previous work indicating a lack of emotion over-responsiveness in participants with SI (see [17]; Ai, et al. [10, 18, 42]), it can be speculated that this emotion regulation inefficiency does not contribute notably to SI risk via enabling heightened emotional responsiveness (which does not seem to be occurring). Instead, it may reflect an increased SI risk associated with inefficient response control over sub-optimal behavioral choices.

### Supplementary information

Supplementary Material

Supplemental Figure 1

## Data Availability

The data that suppor tthe findings of this study are available from the corresponding author upon reasonable request. The data are not publicly available due to IRB restrictions.
