# Correction: Neural correlates of automatic emotion regulation and their association with suicidal ideation in adolescents during the first 90-days of residential care

**DOI:** 10.1038/s41398-024-02817-y

**Published:** 2024-02-19

**Authors:** Matthew Dobbertin, Karina S. Blair, Joseph Aloi, Sahil Bajaj, Johannah Bashford-Largo, Avantika Mathur, Ru Zhang, Erin Carollo, Amanda Schwartz, Jaimie Elowsky, J. L. Ringle, Patrick Tyler, R. James Blair

**Affiliations:** 1 Center for Neurobehavioral Research, Boys Town National Research Hospital, Boys Town, NE USA; 2 Department of Psychiatry, Indiana University School of Medicine, Indianapolis, IN USA; 3 Department of Cancer Systems Imaging, MD Anderson Cancer Center, Houston, TX USA; 4 Multimodal Clinical Neuroimaging Laboratory, Institute for Human Neuroscience, Boys Town, NE USA; 5 Department of Psychology and Human Development, Peabody College, Vanderbilt University, Nashville, TN USA; 6 USC Stevens Neuroimaging and Informatics Institute, Keck School of Medicine of USC, University of Southern California, Los Angeles, CA USA; 7 Stritch School of Medicine, Loyola University Medical Center, Maywood, IL USA; 8 University of North Dakota, Grand Forks, ND USA; 9 University of Nebraska Department of Psychology, Lincoln, NE USA; 10 Child and Family Translational Research, Boys Town National Research Hospital, Boys Town, NE USA; 11 Child and Adolescent Mental Health Centre, Mental Health Services, Capital Region of Denmark, Copenhagen, Denmark

**Keywords:** Predictive markers, Human behaviour

Correction to: *Translational Psychiatry* 10.1038/s41398-023-02723-9, published online 23 January 2024

In this article, the affiliation details for Author Sahil Bajaj were incorrectly given as “Department of Cancer Systems Imaging, MD Anderson Center, South Campus Research Bldg, Houston, TX, USA”

but should have been “Department of Cancer Systems Imaging, MD Anderson Cancer Center, Houston, TX, USA”

The original article has been corrected.

