# Supplementary material for: Neural correlates of automatic emotion regulation and their association with suicidal ideation in adolescents during the first 90-days of residential care
Source: Transl Psychiatry. 2024 Jan 23;14:54. doi: 10.1038/s41398-023-02723-9 (PMC10806086; doi:10.1038/s41398-023-02723-9)
Supplement: Supplementary file 1 — Supplementary Material [file 41398_2023_2723_MOESM1_ESM.docx]

**Supplementary Material**

***Task administration:*** Following the administration of the AS task, participants also received a retaliation task. The data from this task have not been examined with respect to SI and is not reported here.

Supplementary Table S1: Behavioral performance data on the Affective Number Stroop task.

|  | Whole sample | | Participants w/o SI | | Participants w SI | |
| --- | --- | --- | --- | --- | --- | --- |
|  | Mean | SD | Mean | SD | Mean | SD |
| Accuracy |  |  |  |  |  |  |
| NegC | 23.61 | 4.13 | 23.78 | 4.20 | 23.19 | 3.97 |
| NegI | 21.67 | 5.03 | 21.80 | 4.83 | 21.32 | 5.59 |
| NeuC | 23.50 | 4.61 | 23.43 | 4.52 | 23.68 | 4.92 |
| NeuI | 21.35 | 5.38 | 21.19 | 5.48 | 21.77 | 5.19 |
| PosC | 23.04 | 4.70 | 22.99 | 4.54 | 23.16 | 5.15 |
| PosI | 21.86 | 5.24 | 21.99 | 5.23 | 21.55 | 5.33 |
| RT |  |  |  |  |  |  |
| NegC | 769.32 | 198.54 | 767.13 | 202.82 | 774.99 | 190.15 |
| NegI | 848.99 | 188.11 | 844.01 | 187.53 | 861.86 | 192.09 |
| NeuC | 766.35 | 195.48 | 762.25 | 203.65 | 776.92 | 175.30 |
| NeuI | 828.23 | 187.26 | 823.84 | 189.12 | 839.56 | 184.97 |
| PosC | 773.60 | 186.78 | 769.51 | 193.64 | 784.14 | 170.35 |
| PosI | 844.94 | 181.59 | 834.34 | 179.17 | 872.29 | 187.90 |

Key to Table S1: w/o & w=without and with, SI=Suicidal ideation, SD=standard deviation, Neg=Negative, Neu=Neutral, Pos=Positive, C=Congruent, I=Incongruent. Coordinates from the Tournoux and Talairach standard brain template (TT_N27).

Supplementary Table S2: Regions showing significant main effects of Condition and Valence from the 2 (Group)-by-3 (Condition)-by-3 (Valence) repeated measures ANOVA. Activations are effects observed in whole brain analyses significant at p<0.001, corrected for multiple comparisons (significant at p<0.05).

|  | **BA** | **Voxels** | **X** | **Y** | **Z** |
| --- | --- | --- | --- | --- | --- |
|  |  |  |  |  |  |
| **Valence**  R/L Ventromedial frontal cortex | 10/32 | 60 | 5 | 41 | -7 |
| R IFG | 46 | 183 | 53 | 29 | 17 |
| R IFG | 47 | 25 | 26 | 32 | -4 |
| L IFG | 47 | 63 | -34 | 32 | -4 |
| L IFG | 47 | 43 | -40 | 11 | 29 |
| R Inferior parietal lobule | 40 | 33 | 53 | -37 | 44 |
| R Superior temporal sulcus | 22 | 195 | 62 | -16 | 5 |
| L Superior temporal sulcus | 22 | 153 | -61 | -13 | 5 |
| L Cuneus | 18 | 78 | -7 | -82 | 20 |
| L Parahippocampal gyrus | 37 | 135 | -25 | -46 | -10 |
| R Parahippocampal gyrus | 36 | 98 | 29 | -37 | -10 |
| R/L Fusiform, bilateral occipital cortex, extending to L amygdala | -- | 3531 | 38 | -37 | -22 |
| R Amygdala | -- | 43 | 23 | -4 | -13 |
| **Task** |  |  |  |  |  |
| R/L Extensive bilateral region of lateral frontal, dorsomedial, parietal, occipital and temporal cortices | -- | 19646 | -28 | -40 | -28 |
| R/L Ventromedial and rostromedial frontal cortex | 32/10/9 | 1137 | -4 | 38 | -1 |
| R Inferior frontal cortex/orbitofrontal cortex | 11 | 19 | 26 | 44 | -7 |
| R IFG | 45 | 203 | 53 | 23 | 17 |
| L IFG | 47 | 402 | -40 | 29 | -1 |
| R Primary motor cortex | 6 | 238 | 5 | -28 | 59 |
| R Primary motor cortex | 4 | 206 | 29 | -28 | 50 |
| R Posterior insula | 13 | 23 | 38 | -16 | 20 |
| L Precentral gyrus | 6 | 54 | -43 | -10 | 35 |
| L Posterior cingulate cortex | 31 | 25 | -7 | -40 | 35 |
| L Precuneus | 29 | 27 | -7 | -49 | 11 |
| L Parahippocampal gyrus | 36 | 174 | -28 | -34 | -10 |
| R Temporoparietal junction | 22 | 198 | 56 | -43 | 8 |
| L Temporoparietal junction extending into anterior Superior temporal sulcus | 39/22 | 536 | -55 | -58 | 23 |
| R Superior Temporal Sulcus | 21 | 63 | 53 | -1 | -13 |
| L Medial temporal pole | 38 | 49 | -31 | 8 | -34 |
| R Amygdala | -- | 67 | 23 | -7 | -13 |

Key to Table S2: R=Right, L=Left. Coordinates from the Tournoux and Talairach standard brain template (TT_N27).

Supplementary Table S3: Regions showing significant Group-by-Condition-by-Valence and Group-by-Condition interactions from the ANOVA including SSRI medication status as an additional group variable. Activations are effects observed in whole brain analyses significant at p<0.001, corrected for multiple comparisons (significant at p<0.05).

| **REGION** | **BA** | **Voxels** | **X** | **Y** | **Z** |
| --- | --- | --- | --- | --- | --- |
| ***SI-by-Condition-by-Valence*** |  |  |  |  |  |
| R Ventromedial frontal cortex | 10 | 22 | 14 | 59 | -7 |
| R dmPFC | 6 | 108 | 2 | 5 | 62 |
| L Superior parietal lobule | 7 | 81 | -16 | -73 | 44 |
| R Superior parietal lobule | 7 | 69 | 20 | -76 | 44 |
| R Superior frontal gyrus | 6 | 55 | 26 | -7 | 62 |
| R Lateral frontal cortex | 8 | 41 | 41 | 32 | 38 |
| L IFG/OFC | 10 | 35 | -31 | 53 | -1 |
| R IFG /OFC | 11 | 23 | 23 | 38 | -7 |
| R Fusiform gyrus | 37 | 20 | 38 | -55 | -19 |
| ***SI-by-Condition*** |  |  |  |  |  |
| R Superior occipital gyrus | 18 | 71 | 23 | -91 | 14 |
| R Fusiform gyrus | 19 | 98 | 29 | -58 | -10 |
| L Inferior occipital gyrus | 18 | 24 | -37 | -79 | -7 |
| L lingual gyrus | 18 | 38 | -7 | -82 | -4 |

Key to Table S3: R=Right, L=Left. Coordinates from the Tournoux and Talairach standard brain template (TT_N27).

Supplementary Table S4: Regions showing significant Group-by-Condition-by-Valence and Group-by-Condition interactions from the ANCOVA including MFQ score as a covariate. Activations are effects observed in whole brain analyses significant at p<0.001, corrected for multiple comparisons (significant at p<0.05).

| **REGION** | **BA** | **Voxels** | **X** | **Y** | **Z** |
| --- | --- | --- | --- | --- | --- |
| ***SI-by-Condition-by-Valence*** |  |  |  |  |  |
| R DmPFC | 6 | 31 | 2 | -1 | 59 |
| R Frontopolar cortex | 10 | 22 | 14 | 59 | -7 |
| R IFG/OFC | 11 | 22 | 23 | 38 | -7 |
| ***SI-by-Condition*** |  |  |  |  |  |
| R Cuneus | 17 | 105 | 14 | -88 | 11 |
| R Cerebellum/fusiform gyrus | -- | 121 | 26 | -70 | -25 |
| L Inferior occipital gyrus | 18 | 44 | -37 | -79 | -7 |
| L lingual gyrus | 18 | 61 | -7 | -82 | -4 |

Key to Table S4: R=Right, L=Left. Coordinates from the Tournoux and Talairach standard brain template (TT_N27).

Supplementary Table S5: Regions showing significant Group-by-Condition-by-Valence and Group-by-Condition interactions from the ANCOVA including SCARED score as a covariate. Activations are effects observed in whole brain analyses significant at p<0.001, corrected for multiple comparisons (significant at p<0.05).

| **REGION** | **BA** | **Voxels** | **X** | **Y** | **Z** |
| --- | --- | --- | --- | --- | --- |
| ***SI-by-Condition-by-Valence*** |  |  |  |  |  |
| R DmPFC | 6 | 18 | 2 | 1 | 59 |
| R Lateral frontal cortex | 8 | 22 | 41 | 32 | 38 |
| R IFG/OFC | 11 | 15 | 22 | 35 | -8 |
| ***SI-by-Condition*** |  |  |  |  |  |
| R Cuneus | 17 | 81 | 14 | -88 | 11 |
| R Cerebellum/fusiform gyrus | 19 | 90 | 26 | -70 | -25 |
| L Inferior occipital gyrus | 18 | 27 | -37 | -79 | -7 |
| L Lingual gyrus | 18 | 39 | -7 | -82 | -4 |

Key to Table S5: R=Right, L=Left. Coordinates from the Tournoux and Talairach standard brain template (TT_N27).

Supplementary Table S6: Correlations between prescription of anti-psychotic, stimulant and SSRI medications and both SI incidents and scores on the total SRS and its subscales.

|  | Anti-psychotic | Stimulants | SSRI |
| --- | --- | --- | --- |
| SI | 0.077 | -0.131 | -0.049 |
| Total SRS | 0.206 | 0.067 | 0.539^**^ |
| Hopelessness (SRS) | 0.247 | 0.051 | 0.553^**^ |
| Suicidal ideation (SRS) | 0.113 | 0.065 | 0.482^*^ |
| Negative self-evaluation (SRS) | 0.297 | -0.002 | 0.236 |
| Hostility (SRS) | 0.019 | 0.120 | 0.470^*^ |

Key to Table S6: SRS: Suicide Risk Scale, * p<0.05, ** p<0.01.
