# Supplementary material for: Neural correlates of automatic emotion regulation and their association with suicidal ideation in adolescents during the first 90-days of residential care
Source: Transl Psychiatry. 2024 Jan 23;14:54. doi: 10.1038/s41398-023-02723-9 (PMC10806086; doi:10.1038/s41398-023-02723-9)

FIGURE LEGENDS

Supplemental Figure 1. **Task illustration.** Example of **(a)** Negative Incongruent, **(b)** Negative Congruent, and **(c)** Negative View trial.

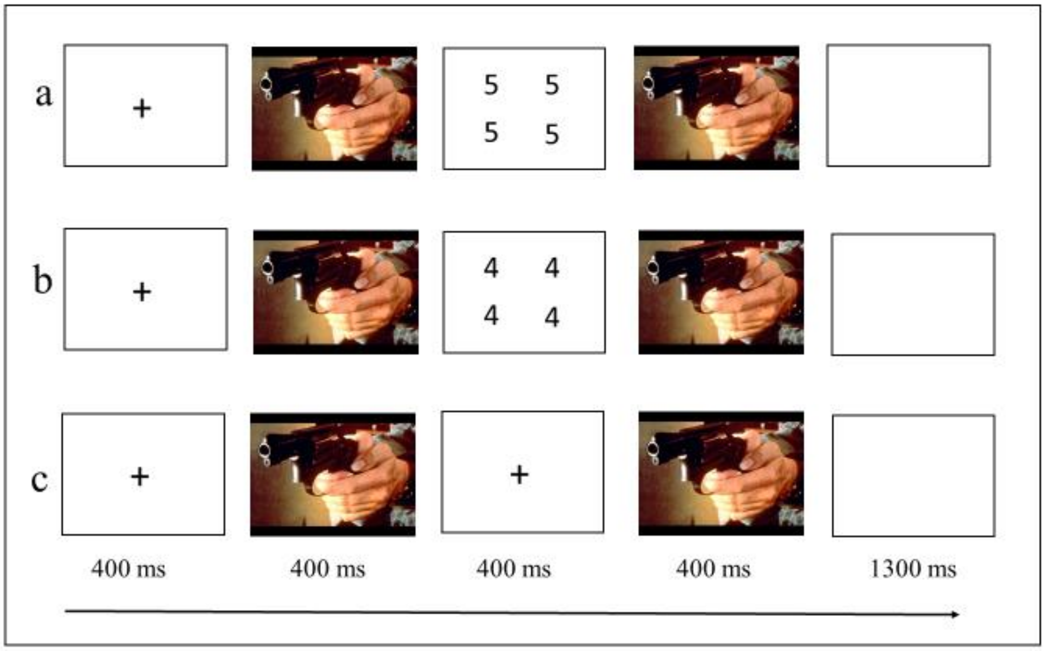

Supplement: Supplementary file 2 — Supplemental Figure 1 [file 41398_2023_2723_MOESM2_ESM.pdf]
